# Gold nanozyme-based paper chip for colorimetric detection of mercury ions

**DOI:** 10.1038/s41598-017-02948-x

**Published:** 2017-06-05

**Authors:** Kwi Nam Han, Jong-Soon Choi, Joseph Kwon

**Affiliations:** Biological Disaster Analysis Group, Korea Basic Science Institute, Daejeon, 169-148 Korea

## Abstract

In this study, we developed a facile gold nanozyme-based paper chip (AuNZ-PAD) for Hg^2+^ detection. This device has the advantages of being simple, rapid, cost effective, sensitive, selective, high throughput, and applicable to onsite detection. The colorimetric mercury assay on the AuNZ-PAD is established based on the enzyme-like catalytic activity of gold nanoparticles promoted by the formation of Au–Hg amalgam, which is correlated to the intensity of the colorimetric response resulting from the catalytic reaction of 3,3′,5,5′-tetramethylbenzidine (TMB) and H_2_O_2_. Highly sensitive and selective detection of Hg^2+^ ions is achieved in both distilled and tap water samples, indicating the feasibility and applicability of our device for the determination of mercury pollution in real samples. Moreover, AuNZ-PAD analysis using a smartphone camera eliminates the need for expensive analytical equipment, thereby increasing the practicality of field monitoring of trace Hg^2+^ compared with other sensing methods.

## Introduction

Mercury is one of the most common toxic heavy metals in the environment and is widely found in water, soil, and even food. The amount mercury released globally from human activities (power plants, waste incineration, and metal mining) and natural events is estimated to be 5,000–8,000 metric tons per year^1^. Exposure to mercury ions causes serious damage to the organs and immune system of humans, even at low concentrations, and its accumulation in the body can result in several diseases (acrodynia, Hunter–Russell syndrome, and Minamata disease), permanent damage to the brain, kidneys, and nervous system, and even death^2^. Owing to the acute toxicity of mercury, the World Health Organization (WHO) and the U.S. Environmental Protection Agency (EPA) have set maximum allowable levels of mercury in drinking water at 6 and 2 μg L^−1^, respectively^3^. At present, traditional methods used for Hg^2+^ detection include inductively coupled plasma mass spectrometry (ICP-MS) and atomic absorption/emission spectrometry (AAS/AES). These spectroscopic techniques offer high sensitivity and selectivity, but are expensive (~$50 per sample) and require sophisticated equipment, trained personnel, and time-consuming sample preparation, which limit their practical application for onsite analysis.

To overcome these drawbacks, a variety of nanomaterials have been utilized to develop colorimetric, fluorescent, and electrochemical sensors for trace Hg^2+^ detection in the field^4–7^. Common detection strategies for selective and sensitive analysis involve surface modification of nanomaterials, such as oligonucleotides^6–9^, proteins/peptides^10^, aptamers^11^, thiol compounds^12, 13^, and cysteine^14^, allowing specific interaction with Hg^2+^ and signal transduction depending on Hg^2+^ concentration. Moreover, some new approaches have been developed for Hg^2+^ detection using several nanomaterials (e.g., gold, platinum, and graphene oxide) based on their unique catalytic properties^15–17^. These nanomaterials, called nanozymes, exhibit intrinsic enzyme-mimetic activity similar to that of natural peroxidases (e.g., horseradish peroxidase, HRP), which can catalyze H_2_O_2_-mediated oxidation of peroxidase substrates or Amplex UltraRed^18^. Consequently, changes in the colorimetric or fluorogenic response of the solution are correlated to Hg^2+^ concentration. Although these nanomaterial-based sensors have demonstrated excellent analytical performance for Hg^2+^ detection, they suffer from expensive and complicated surface modifications and require sophisticated analytical instruments for readouts (fluorescence/absorption spectrophotometer or electrochemical analyzer). Therefore, the development of simple, cheap, sensitive, and selective sensors for onsite monitoring of mercury levels remains challenging.

In recent years, paper-based analytical devices (PADs) have received considerable attention for point-of-care testing and field monitoring owing to the advantages of low cost, light weight, ease of handling (e.g., folding, cutting, and patterning), and low sample/reagent consumption^19, 20^. Most PADs are fabricated by a simple wax printing method to create hydrophilic/hydrophobic patterns for fluid transport via capillary action, and are commonly used for colorimetric analysis owing to the high color contrast on paper substrates^21^. The assay results can be directly interpreted by the naked eye and quantified using a mobile camera instead of bulky instruments, making PADs ideal for onsite monitoring applications^22, 23^.

Herein, we present a gold nanozyme-based paper chip (AuNZ-PAD) for simple, rapid, sensitive, selective, and cost-effective detection of trace Hg^2+^ in aqueous systems. Gold nanoparticles (AuNPs) were employed as a sensitive and selective probe for colorimetric assays based on their enzyme-like catalytic activity, which could be enhanced remarkably by the formation of Au–Hg amalgam (alloy). The chromogenic enzyme substrates were catalytically oxidized on Au–Hg amalgam in the presence of H_2_O_2_, resulting in a blue stain on the paper chip. The mercury-promoted nanozyme activity of AuNPs was systemically investigated under various conditions (AuNP size, H_2_O_2_ concentration, and pH). The analytical performance of the AuNZ-PAD was demonstrated in both distilled and tap water samples. Moreover, coupling this paper chip with a smartphone camera eliminated the need for expensive, sophisticated equipment for readouts.

## Results

**Figure 1:**
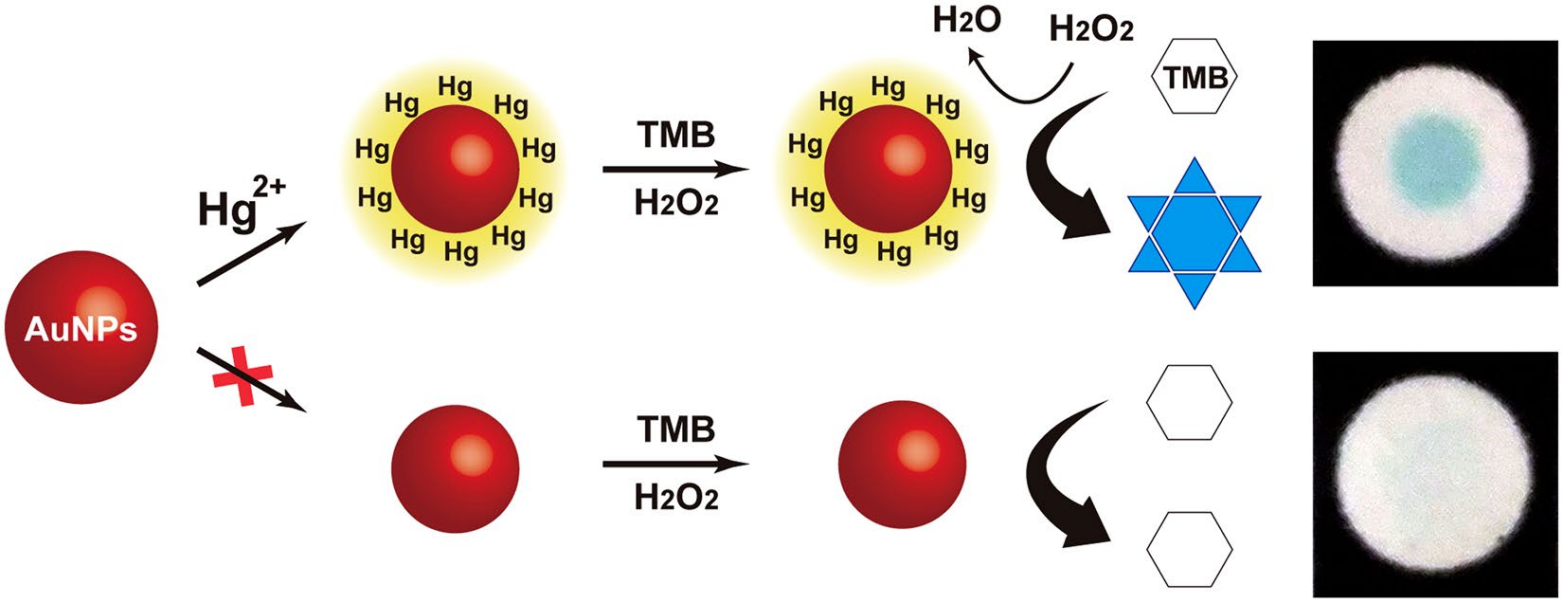
Schematic illustration of the AuNZ-PAD colorimetric sensing mechanism for Hg^2+^ ions based on the mercury-promoted nanozyme activity of AuNPs. When Hg^2+^ ions are introduced onto the AuNZ-PAD, the TMB–H_2_O_2_ catalytic reaction is highly enhanced by the formation of Au–Hg amalgam, resulting in blue staining of the paper chip.

It is known that several nanomaterials (e.g., gold, platinum, graphene oxide, Fe_3_O_4_, and CeO_2_) possess intrinsic peroxidase-like catalytic activity^24–32^. Among these materials, citrate-stabilized AuNPs exhibit relatively low catalytic activity^33^. However, it was recently reported that the nanozyme activity of AuNPs can be enhanced considerably by the formation of Au–Hg amalgam in aqueous solution^34^. By taking advantage of the mercury-promoted nanozyme activity of AuNPs, colorimetric mercury detection was established, as depicted in Fig. 1. When Hg^2+^ ions are introduced on the AuNZ-PAD, they are spontaneously deposited on the surface of AuNPs, resulting in the formation of Au–Hg amalgam by specific metallophilic interactions^35, 36^. The presence of Hg atoms around AuNPs causes changes in the surface properties of AuNPs, which accelerate decomposition of H_2_O_2_, leading to considerable improvement of the peroxidase-like catalytic activity of AuNPs^34, 37^. As a result, an intense blue stain is produced on the paper chip by the catalytic reaction of TMB (chromogenic peroxidase substrate) with H_2_O_2_. In contrast, the TMB–H_2_O_2_ catalytic reaction hardly occurs on bare AuNPs owing to their low catalytic activity, and no color change is observed on the paper chip.

To demonstrate the mercury-promoted nanozyme activity of AuNPs, the TMB–H_2_O_2_ catalytic reaction was investigated using UV–vis spectroscopy. As shown in Supplementary Figure S1, negligible color change was observed in the absence and presence of AuNPs (curves a and b), indicating the poor nanozyme activity of bare AuNPs. However, when AuNPs and Hg^2+^ ions coexisted in solution, the TMB–H_2_O_2_ catalytic reaction proceeded to produce oxidized TMB, which has an intense blue color in solution, with a maximum absorbance at 652 nm (curve c). This result reveals that the TMB–H_2_O_2_ catalytic reaction is accelerated in the presence of both AuNPs and Hg^2+^ ions, suggesting that the nanozyme activity of AuNPs can be enhanced when Au–Hg amalgam is formed by the addition of Hg^2+^ ions. In addition, the Au–Hg amalgam was characterized by X-ray photoelectron spectroscopy (XPS) and transmission electron microscopy (TEM). The Au 4 f XPS spectrum shows two peaks at 84.1 and 87.8 eV corresponding to Au 4f_7/2_ and Au 4f_5/2_, respectively which were assigned to metallic Au^0^ (Supplementary Figure S2A)^38^. In Supplementary Figure S2B, the two peaks observed in the Hg 4 f XPS spectrum at 99.8 eV (Hg 4f_7/2_) and 103.8 eV (Hg 4f_5/2_) correspond to the Hg^0^ oxidation state. These results reveal that metallic mercury was indeed formed on the surface of the AuNPs^39^. Furthermore, TEM images of the AuNPs in Supplementary Figure S3 show no obvious difference in the particle size of the AuNPs before and after addition of Hg^2+^ ions, indicating that only a monolayer or submonolayer of Hg^0^ was formed on the surfaces of the AuNPs.

**Figure 2:**
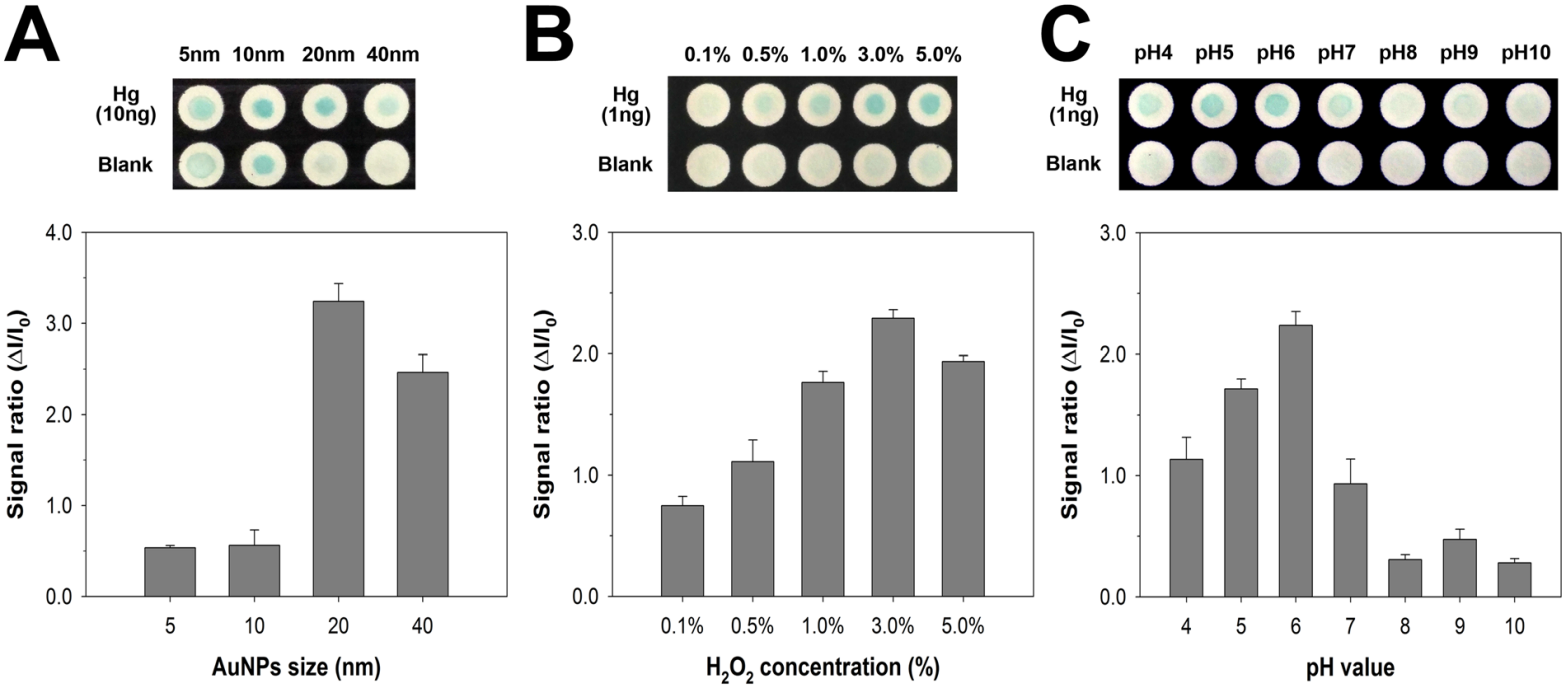
Effect of (**A**) the size of AuNPs, (**B**) the concentration of H_2_O_2_, and (**C**) the pH value on the colorimetric response in the absence and presence of Hg^2+^ ions. Error bars indicate the standard deviation (SD) of three independent experiments.

To study the effect of experimental parameters (AuNP size, H_2_O_2_ concentration, and pH) on the mercury-promoted nanozyme activity of AuNPs, we systemically investigated the colorimetric response on paper in the absence and presence of Hg^2+^ ions under various conditions. Figure 2A shows the effect of AuNP size on the colorimetric response of the TMB–H_2_O_2_ catalytic reaction. For the tests, 1 μL of AuNP solution (OD of 1 at 520 nm) were drop-dried on each test zone with a diameter of 10 mm. Experiments were then carried out by adding 1 μL of the test sample, followed by the addition of 2 μL of TMB substrate containing 0.5% H_2_O_2_. The AuNP size was varied from 5 to 40 nm, and the size dependence of the mercury-promoted nanozyme activity of AuNPs was evaluated by comparing the colorimetric intensity ratio, Δ*I*/*I*_0_ = (*I* − *I*_0_)/*I*_0_, where *I* and *I*_0_ denote the colorimetric intensity values obtained for 10 ng Hg and a blank solution, respectively. As shown in Fig. 2A, 20 nm AuNPs showed the highest sensitivity to Hg^2+^, with a significant difference in color observed, whereas AuNPs with sizes below 10 nm exhibited a blue stain irrespective of the addition of Hg^2+^ owing to strong intrinsic catalytic activities induced by the high surface-to-volume ratio of small particles. These results demonstrated that the AuNP size has an important impact on mercury-promoted nanozyme activity, and 20 nm AuNPs are the most appropriate for Hg^2+^ detection.

Figure 2B and C show the effect of H_2_O_2_ concentration and pH on the colorimetric response, respectively. For these experiments, 20 nm AuNPs (OD of 1/6, 0.194 nM) were applied as the optimum condition. As shown in Fig. 2B, the colorimetric signal ratio gradually increased upon increasing the H_2_O_2_ concentration up to 3.0%, as oxidation of TMB is established in the presence of H_2_O_2_. However, further increasing the concentration decreased the signal ratio, indicating that the optimal H_2_O_2_ concentration for the TMB–H_2_O_2_ catalytic reaction on AuNZ-PAD is 3.0%. Moreover, to examine the pH dependence of the catalytic activity, the Hg^2+^ samples were diluted with 10 mM acetate buffer in the pH range of 4.0–10.0, followed by application of the TMB substrate containing 3.0% H_2_O_2_. The maximum mercury-promoted nanozyme activity of AuNPs occurred at approximately pH 6.0 (Fig. 2C). The colorimetric signal ratio increased with increasing pH from 4.0 to 6.0, and then decreased drastically when the pH value exceeded 7.0. At high pH conditions, mercury oxide/hydroxide species (HgO and Hg(OH)_2_) could be formed through interactions with OH^−^ ions, which would prevent the formation of Au–Hg amalgam, resulting in reduction of the nanozyme activity of AuNPs for the TMB–H_2_O_2_ catalytic reaction^40, 41^. In addition, the optimum pH for peroxidase-catalyzed oxidation of TMB substrate is typically between 4.0 and 7.0^42^. Therefore, based on these results, further tests on paper chips were performed under the optimized conditions (20 nm AuNPs, 3.0% H_2_O_2_, and pH 6.0).

**Figure 3:**
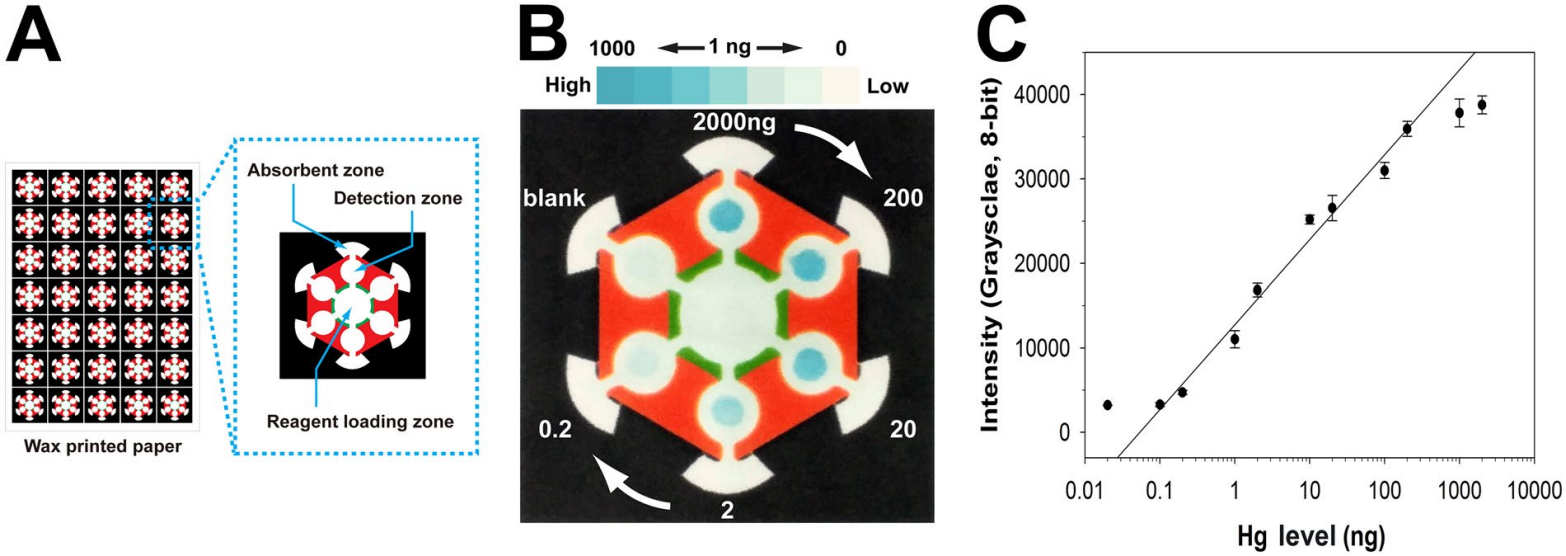
(**A**) Illustration of the AuNZ-PAD and its component parts. The white and colored (black, red, and green) regions represent the hydrophilic paper and hydrophobic wax, respectively. (**B**) Photographic image of an AuNZ-PAD used to simultaneous test samples with Hg levels ranging from 0.2 to 2000 ng. (**C**) Calibration plot of colorimetric responses for Hg levels in the range of 0.02–2000 ng. Error bars represent the SD of three independent experiments.

The design of the AuNZ-PAD is shown in Fig. 3A. The AuNZ-PAD consists of three zones: reagent loading, detection, and absorbent. To perform the colorimetric assay on the AuNZ-PAD, Hg^2+^ samples diluted with 10 mM acetate buffer (pH 6.0) were applied on the detection zone, which includes six test zones where 20 nm AuNPs were previously integrated, allowing simultaneous analysis of multiple samples. Subsequently, TMB substrate containing 3.0% H_2_O_2_ was added on the reagent loading zone. The TMB substrate then spread to the six test zones by capillary action until reaching the arch-shaped absorbent zones. After 10 min, the colorimetric assay results could be readily observed by the naked eye. Figure 3B shows a photographic image of the AuNZ-PAD taken using a smartphone camera. Six samples with different mercury level were simultaneously tested on a single paper chip. As the mercury level increased, the color of the test zone changed from colorless to dark blue, which indicated that the intensity of color corresponds to the amount of mercury in the sample. The calibration plot in Fig. 3C shows a good linear relationship between the colorimetric responses and the logarithmic values of the mercury levels in the range between 0.1 and 200 ng. The colorimetric signal intensity was saturated above 500 ng Hg, which may be due to the limited amount of AuNPs available for forming Au–Hg amalgam. These results demonstrated that the AuNZ-PAD provides a large linear dynamic range (about three orders of magnitude) for Hg^2+^ detection, which is an important factor for quantitative analysis in practical applications.

**Figure 4:**
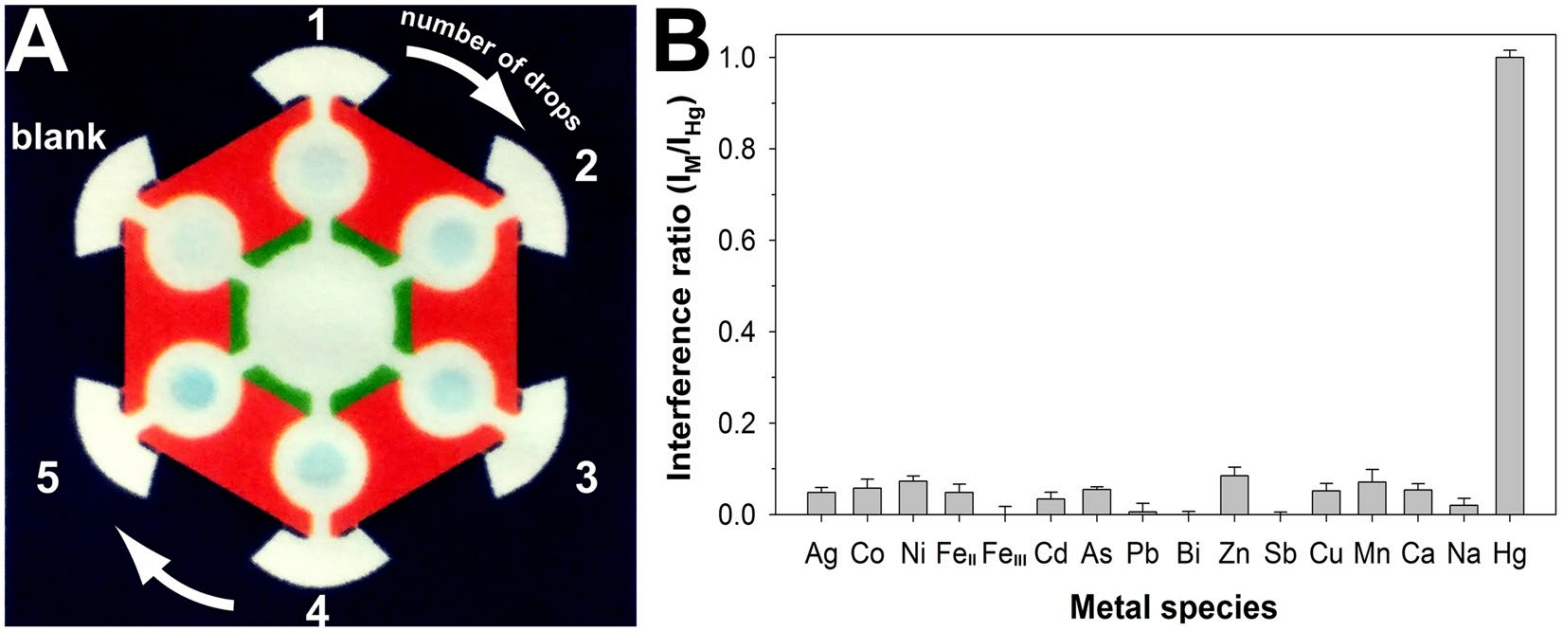
(**A**) Colorimetric signal amplification depending on the number of drops of test sample. A 2 μL drop of test sample containing 0.4 ng Hg was repetitively applied on each test zone of the AuNZ-PAD with a time interval of 2 min. (**B**) Selectivity of the AuNZ-PAD against other metal/metalloid species. The added levels of Hg^2+^ and other metal/metalloid species were 1 and 100 ng, respectively. Error bars represent the SD of three independent experiments.

Figure 4A shows an interesting feature of the AuNZ-PAD for colorimetric signal amplification. In this experiment, a 2 μL drop of test sample containing 0.4 ng Hg was repetitively applied on each test zone of the AuNZ-PAD with a time interval of 2 min. As the number of drops of test sample increased, a conspicuous color change was observed, as shown in Fig. 4A. Moreover, the colorimetric signal intensity was found to be proportional to the number of drops (Supplementary Figure S4). These results revealed that multiple applications of test sample allow for *in situ* preconcentration of trace Hg^2+^ on the AuNZ-PAD, thereby leading to an enhancement of the colorimetric response. Therefore, it is reasonable to expect that the detection sensitivity and dynamic range of this device can be easily improved by repeated additions of test sample. Considering the preconcentration factor, the linear regression equation can be expressed as *I* = 10049 log[*n* Hg(ng)] + 12780, where *n* corresponds to the number of drops of test sample, with a correlation coefficient of 0.986. The limit of detection (LOD, 3 SD) was estimated to be 0.06 ng (30 μg L^−1^) for a single drop of test sample, and could be reduced to 0.012 ng (1.2 μg L^−1^) by using five applications of test sample. Moreover, if needed, the LOD can be further decreased by simply increasing the number of drops of test sample. Thus, our device offers an LOD that is low enough for drinking water analysis and has a wider dynamic range (>10-fold) than other paper-based sensors, as shown in Supplementary Table S1. Although ICP-MS analysis provides an excellent LOD (0.001 μg L^−1^) and a broad dynamic range (8–9 orders of magnitude) compared with those of paper-based sensors, it requires expensive instrumentation and highly trained operators, and suffers from severe memory effects owing to adsorption of mercury. Therefore, our paper chip is potentially more applicable for on-site mercury analysis because of its low cost, rapid response, portability, and low labor requirement.

To demonstrate the selectivity of the AuNZ-PAD toward Hg^2+^, 15 other metal/metalloid ions (Ag^+^, Fe^2+^, Fe^3+^, Ni^2+^, Co^2+^, Cd^2+^, As^3+^, Pb^2+^, Bi^3+^, Zn^2+^, Sb^3+^, Cu^2+^, Mn^2+^, Ca^2+^, and Na^+^) were tested. Figure 4B and Supplementary Figure S5 show the colorimetric responses against metal/metalloid species (100 ng) and mercury (1 ng). Although the amounts of the interfering species were 100-times greater than that of mercury, only Hg^2+^ ions caused a conspicuous color change of the test zone and negligible colorimetric responses were observed for each of the interfering species (less than 8% of that of Hg^2+^; Fig. 4B). Thus, our device showed excellent selectivity toward Hg^2+^ over other interfering species owing to the amalgam process that occurs specifically between Au and Hg atoms.

To further demonstrate the applicability of the AuNZ-PAD in practical applications, different concentrations of Hg^2+^ (0.05, 0.5, 5, and 50 mg L^−1^) were spiked in tap water (1:1 mixed with 10 mM acetate buffer, pH 6.0) and tested on our device. Tap water was collected from the laboratory in our institute. As summarized in Table 1, the obtained recoveries were in the acceptable range of 84.4–112.9% with a relative standard deviation (RSD) of 3.8–8.7% for four different Hg^2+^ spiked levels in tap water. The results clearly demonstrate that the AuNZ-PAD is applicable for practical analysis of Hg^2+^ in real samples.

**Table 1:** Determination of Hg^2+^ in tap water samples using the AuNZ-PAD.

Sample	Hg^2+^ added (mg L^−1^)	Mean detected (mg L^−1^)	Recovery (%)	RSD (%)
1	0.05	0.053	105.9	6.3
2	0.5	0.422	84.4	3.8
3	5	5.65	112.9	8.6
4	50	48.22	96.4	8.7

*Mean values and RSDs were obtained from three independent experiments.

## Discussion

We report a new paper-based mercury sensor based on the nanozyme activity of AuNPs. This device provides a simple, portable, and cost-effective approach for the determination of Hg^2+^ without requiring complicated and time-consuming surface modification with Hg^2+^-specific ligands and bulky and expensive instruments for readouts. Moreover, multiple tests can be simultaneously performed on a single device and readily analyzed using a smartphone camera. The AuNZ-PAD showed high sensitivity with a wide detection dynamic range (about three orders of magnitude) and selective specificity toward Hg^2+^ without interference from other metal/metalloid species. The LOD for Hg^2+^ was found to be 0.06 ng and 0.012 ng for a single application and five applications of test sample, respectively, and could be further improved by repeated additions of test sample. These results suggest that the proposed AuNZ-PAD is capable of facilitating onsite monitoring of mercury pollution in aqueous environments for the prevention of mercury poisoning.

## Methods

### Materials and instruments

Chromatography paper (grade 1 cellulose) was purchased from GE Healthcare (Piscataway, NJ, USA). Colloidal gold solutions (citrate stabilized) with particles diameters of 5, 10, 20, and 40 nm were obtained from Sigma-Aldrich (St. Louis, MO, USA) and Median Diagnostics Inc. (Chuncheon, South Korea). 3,3′,5,5′-Tetramethylbenzidine (TMB) substrate was purchased from Cell Signaling Technology Inc. (Danvers, MA, USA). Metal/metalloid ions (Ag^+^, Fe^2+^, Fe^3+^, Ni^2+^, Co^2+^, Cd^2+^, As^3+^, Pb^2+^, Bi^3+^, Zn^2+^, Sb^3+^, Cu^2+^, Mn^2+^, Ca^2+^, Na^+^, and Hg^2+^) were obtained from Sigma-Aldrich (St. Louis, MO, USA). All other chemicals were of analytical grade, and the chromatography paper and all chemicals were used without any pretreatment or purification. Aqueous solutions were prepared using deionized water. The absorbance of the solution was determined using a UV–visible spectrophotometer (ProteomeLab Du-800, Beckman Coulter, Inc., Fullerton, CA, USA). TEM measurements were preformed using a JEM-2100F instrument (JEOL, Tokyo, Japan) at an acceleration voltage of 200 kV. XPS measurements were carried out using an Axis Nova spectrometer (Kratos Analytical Ltd., Manchester, UK) with a monochromatic Al Kα source (1486.6 eV).

### Fabrication of AuNZ-PAD

Paper-based devices were fabricated using a commercially available wax printer (Color Cube 8870, Xerox, Norwalk, CT, USA) as previously reported^43^. Hydrophobic patterns, which were designed using graphics software (Adobe Illustrator CS3), were printed on chromatography paper to define the fluidic path and control liquid transport. The paper was heated at 100 °C for 1 min in a dry oven to melt the patterned wax. The patterned paper (6.0 × 6.0 cm) was cut, and the backside of each piece of paper was fully covered by an adhesive PET film. To integrate AuNPs onto the paper chip, 1.5 μL of 20 nm AuNP solution (0.194 nM) was drop-dried on each test zone (with a diameter of 1.0 cm) at ambient temperature.

### Assay procedure and signal quantification

A 2 μL volume of sample was applied to each test zone of the paper chip. Then, 100 μL of 0.05% TMB solution containing 3.0% H_2_O_2_ was added to the reagent loading zone. This solution then flowed along the fluidic path until it reached the arch-shaped absorbent zones. The assay results were directly visible to the naked eye, amplifying over time. To quantify the results, after 10 min incubation, photographs were taken using a smartphone camera (LG-G5, 16.0 megapixels) with a shutter speed of 1/20 at ISO 50. Subsequently, colorimetric signal analysis was carried out using ImageJ software (National Institutes of Health, Bethesda, MD, USA).

## Electronic supplementary material

Supplementary Information
