# Supplementary material for: Gold nanozyme-based paper chip for colorimetric detection of mercury ions
Source: Sci Rep. 2017 Jun 5;7:2806. doi: 10.1038/s41598-017-02948-x (PMC5459837; doi:10.1038/s41598-017-02948-x)
Supplement: Supplementary file 1 — Supplementary Information [file 41598_2017_2948_MOESM1_ESM.doc]

**Gold nanozyme-based paper chip for colorimetric detection of mercury ions**

Kwi Nam Han, Jong-Soon Choi* and Joseph Kwon*

Biological Disaster Analysis Group, Korea Basic Science Institute, Daejeon 169-148, Korea

Corresponding authors:

Tel.: +82 42 865 3446

E-mail: joseph@kbsi.re.kr (J.K.) and jschoi@kbsi.re.kr (J.-S.C.)

**Supplementary Information**


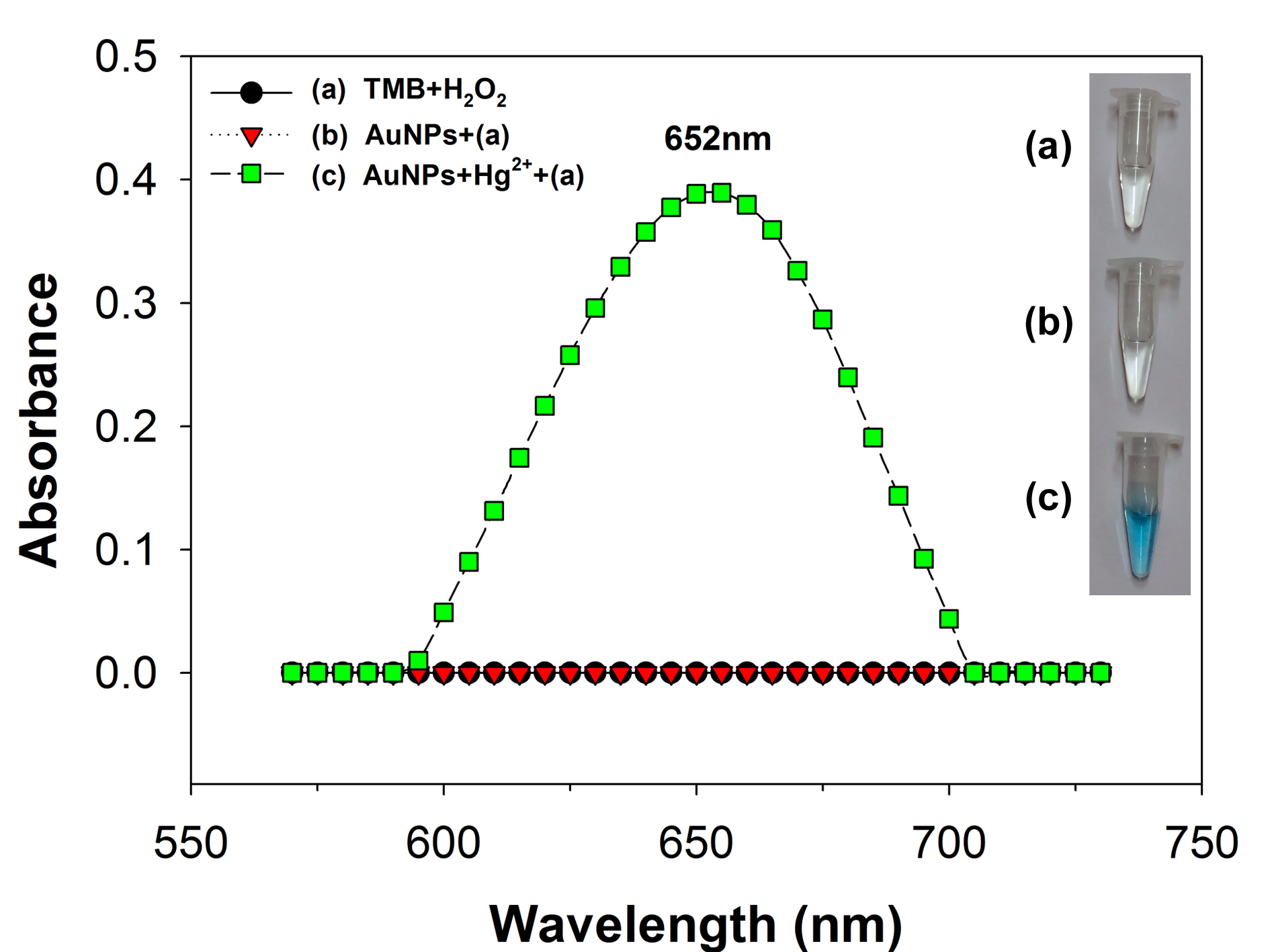


**Figure S1.** UV–vis spectra of (a) TMB solution with 0.5% H_2_O_2_; (b) solution (a) after the addition of 10 μL of AuNPs (1.16 nM, 20 nm); (c) solution (a) after the addition of 10 μL each of AuNPs (1.16 nM, 20 nm) and Hg^2+^ (100 mg L^-1^). Data were collected after 10 min of incubation. The inset shows corresponding photographic images of (a)–(c).


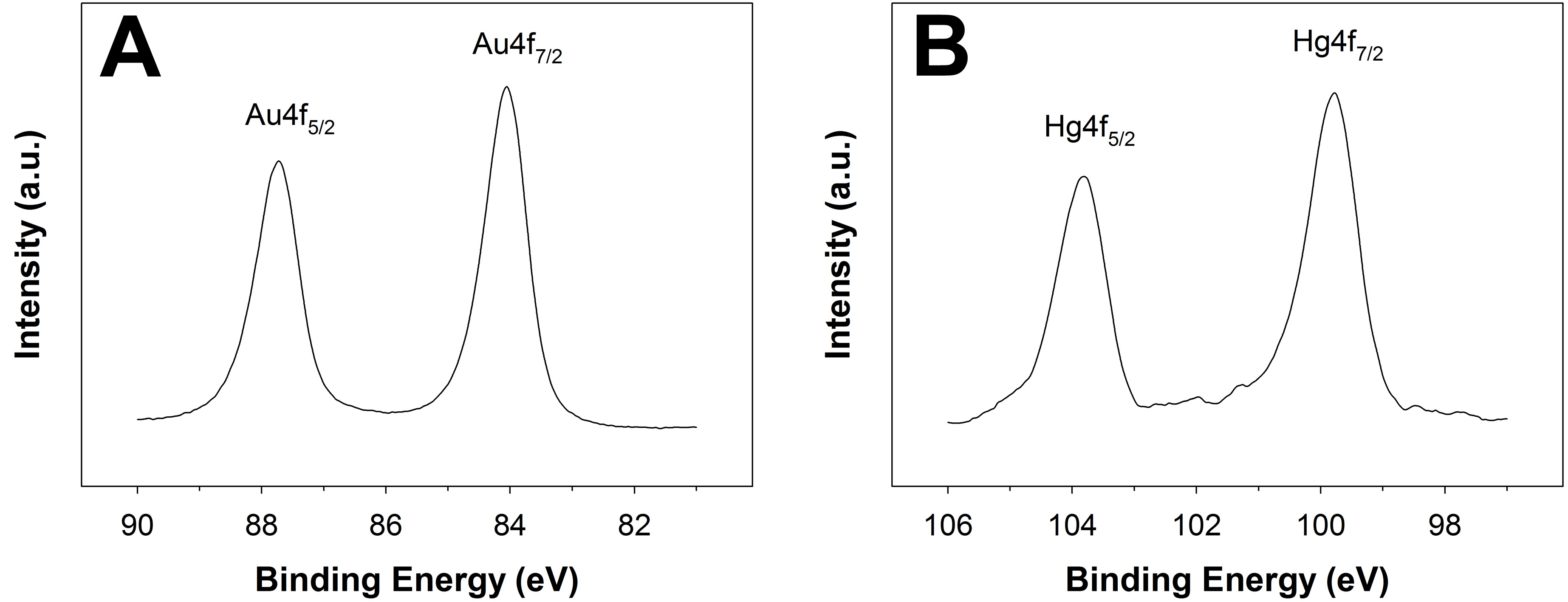


**Figure S2.** XPS analysis of Au–Hg amalgam. (A) Au 4f core level spectrum. (B) Hg 4f core level spectrum.


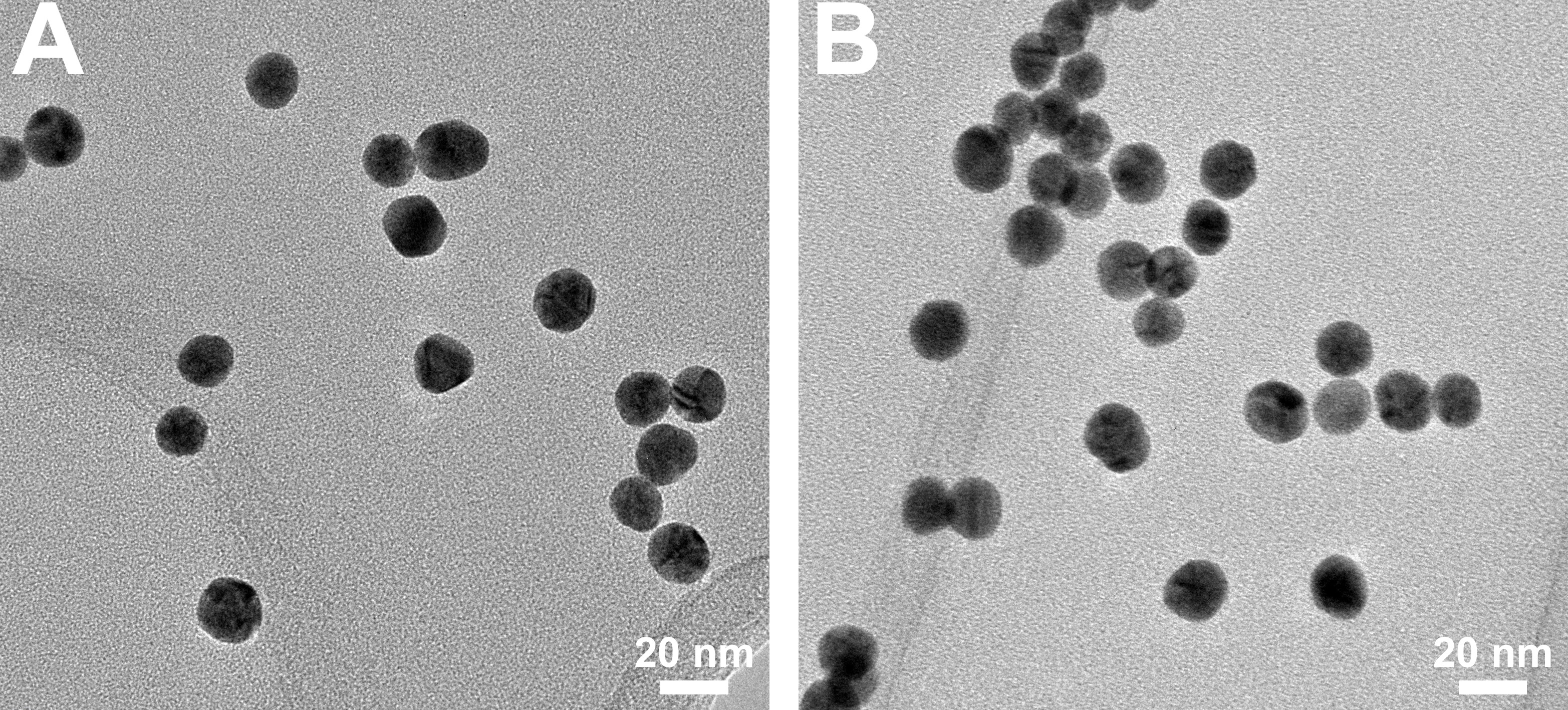


**Figure S3.** TEM images of AuNPs in the absence (A) and presence (B) of Hg^2+^ ions.


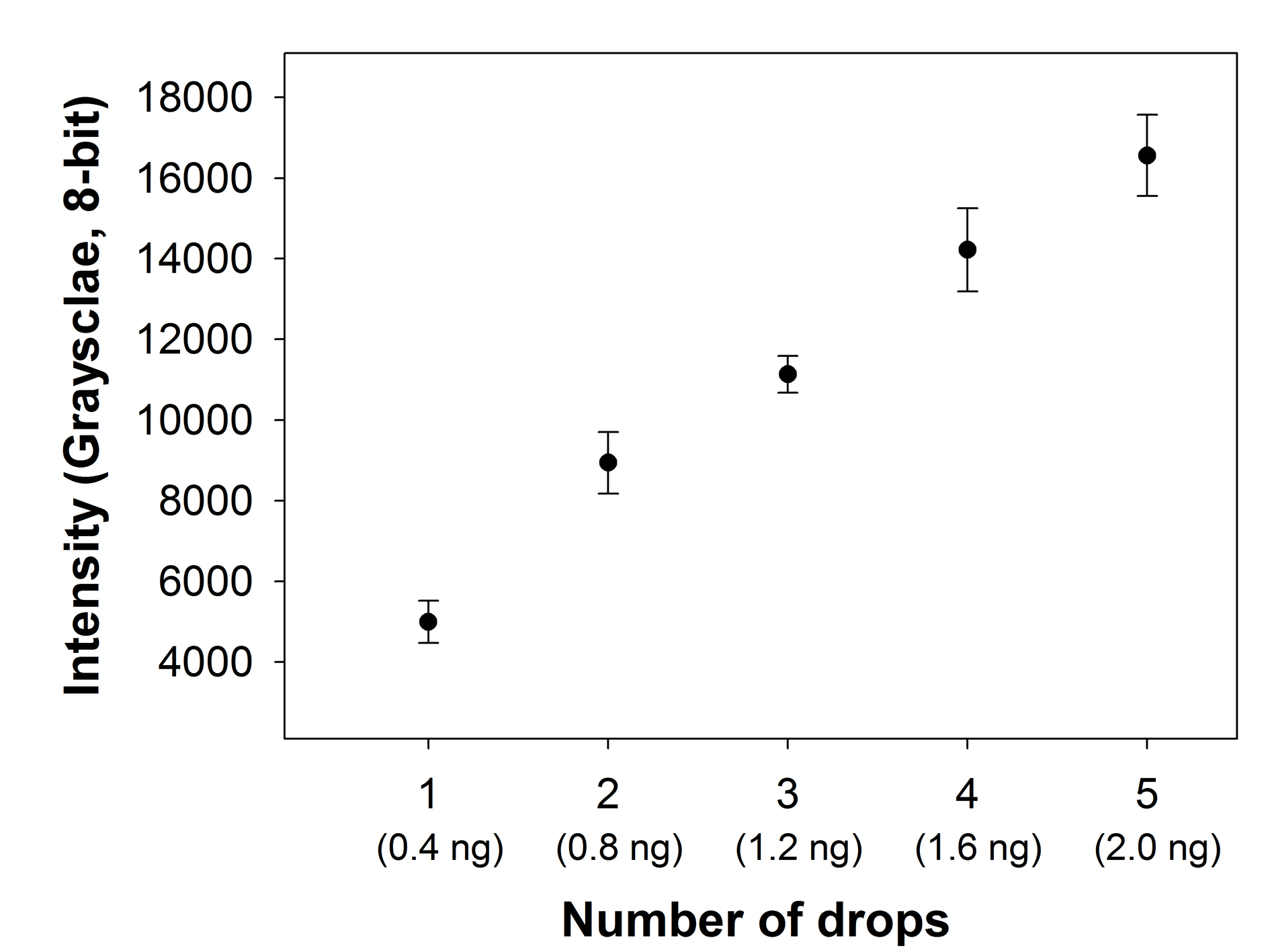


**Figure S4.** Effect of the number of drops on colorimetric signal amplification. The resulting intensities are proportional to the number of test sample drops applied. The corresponding Hg levels are given in parentheses. Error bars represent the SD of three independent experiments.


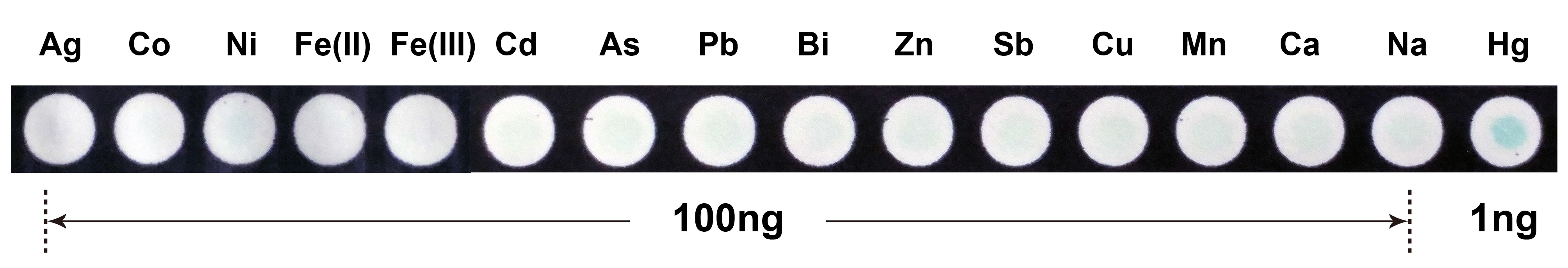


**Figure S5.** Photographic image showing the colorimetric responses in the presence of mercury (1 ng) and interfering metal/metalloid species (100 ng).

**Table S1** Comparison of different types of paper-based sensors for Hg^2+^ detection.

| Detection method | Sensing probe/modifier*^a^* | Linear range  (mg L^-1^) | LOD  (μg L^-1^) | Ref. |
| --- | --- | --- | --- | --- |
| Colorimetry | AuNPs | 0.05–100 | 30  (1.2 at *n^b^* = 5) | This work |
| Colorimetry | Chromogenic complexing agent | 10–400 | 10 | 1 |
| Colorimetry | ssDNA/AuNPs | 0.005–0.02 | 10 | 2 |
| Colorimetry | PtNPs | 5–100 | 2 | 3 |
| Colorimetry | AgNPls | 5–25 | 120 | 4 |
| Fluorescence | Cy5/ssDNA/GO | <0.6 | 24 | 5 |
| Fluorescence | Fluorogenic complexing agent | 0.02–0.2 | 20 | 6 |
| Electrochemistry | PPy/Cellulose | >1 | 200 | 7 |
| Electrochemistry | PEG-SH/SePs/AuNPs | 0.014–3.5 | 1 | 8 |

*^a^* AuNPs, gold nanoparticles; ssDNA, single-stranded DNA; PtNPs, platinum nanoparticles; AgNPls, silver nanoplates; GO, graphene oxide; PPy, polypyrrole; PEG-SH, poly(ethylene glycol) methyl ether thiol; SePs, selenium particles

*^b^* Number of drops of test sample

**Supplementary References**

1. Das, P., Ghosh, A., Bhatt, H., Das, A. A highly selective and dual responsive test paper sensor of Hg^2+^/Cr^3+^ for naked eye detection in neutral water. *RSC Adv.* **2**, 3714–3721 (2012).
2. Chen, G. H. *et al.* Detection of mercury(II) ions using colorimetric gold nanoparticles on paper-based analytical devices. *Anal. Chem.* **86**, 6843–6849 (2014).
3. Chen, W., Fang, X., Li, H., Cao, H., Kong, J. A Simple Paper-Based Colorimetric Device for Rapid Mercury(II) Assay. *Sci. Rep.* **6**, 31948 (2016).
4. Apilux, A., Siangproh, W., Praphairaksit, N., Chailapakul, O. Simple and rapid colorimetric detection of Hg(II) by a paper-based device using silver nanoplates. *Talanta* **97**, 388–394 (2012).
5. Zhang, Y., Zuo, P., Ye, B. C. A low-cost and simple paper-based microfluidic device for simultaneous multiplex determination of different types of chemical contaminants in food. *Biosens. Bioelectron.* **68**, 14–19 (2015).
6. Hatai, J., Pal, S., Jose, G. P., Bandyopadhyay, S. Histidine based fluorescence sensor detects Hg^2+^ in solution, paper strips, and in cells. *Inorg. Chem.* **51**, 10129–10135 (2012).
7. Lee, J. E., Shim, H. W., Kwon, O. S., Huh, Y. I., Yoon, H. Real-time detection of metal ions using conjugated polymer composite papers. *Analyst* **139**, 4466–4475 (2014).
8. Bui, M. P., Brockgreitens, J., Ahmed, S., Abbas, A. Dual detection of nitrate and mercury in water using disposable electrochemical sensors. *Biosens. Bioelectron.* **85**, 280–286 (2016).
